# Measuring Spontaneous Focus on Space in Preschool Children

**DOI:** 10.3389/fpsyg.2019.02624

**Published:** 2019-11-28

**Authors:** Jasmin Perez, Koleen McCrink

**Affiliations:** ^1^Department of Psychological and Brain Sciences, Johns Hopkins University, Baltimore, MD, United States; ^2^Department of Psychology, Barnard College, Columbia University, New York, NY, United States

**Keywords:** space, proportional reasoning, spontaneous focus on numerosity, spontaneous focus on space, spatial relations

## Abstract

Previous work on children’s Spontaneous Focus on Numerosity (SFON) has shown the value of measuring children’s spontaneous attention within naturalistic interactions. SFON is the spontaneous tendency to focus attention on, and explicitly enumerate the exact number of, items in a set. This measure predicts later math skills above and beyond general IQ and other cognitive factors such as attention. The utility of SFON suggests that a parallel construct for space is a worthy pursuit; spatial cognition underlies many of our mathematical skills, especially as children are first learning these skills. We developed a measure of children’s Spontaneous Focus on Space – the spontaneous tendency to attend to absolute and relative spatial components of the environment – and studied its relation to reasoning about the important spatial-numerical concept of proportions. Fifty-five 3- to 6-year-olds were tested at a local children’s museums in New York City. Children participated in tasks designed to measure their spontaneous focus on space and number, and their ability to reason about spatial proportions. Results indicate that as children grow older, their Spontaneous Focus on Space becomes more complete and is positively related to proportional reasoning performance. These findings suggest that spatial awareness is rapidly increasing in the preschool years, alongside numerical awareness and spatial-numerical proportional reasoning.

From early in development, humans and other animals represent magnitudes in the world around them (Gallistel, 1990; Wynn, 1998; Dehaene, 2007; Feigenson, 2007). These systems for conceptualizing space, number, and time have mental primacy (Dehaene and Brannon, 2011; Newcombe et al., 2015) and combine to influence fundamental processes such as learning and memory. Consequently, attending to the spatial relations of items in the world often implicitly enhances encoding and memory. For example, in visual search tasks, when target objects are presented in previously seen configurations (i.e., a target’s location and its spatial relations to others are held constant), they are more rapidly found than when displayed in novel contexts (Chun and Jiang, 1998). Importantly, the observed facilitation of such *contextual cuing* is implicit; although it facilitates visual search, identification of the repeated configurations is not enhanced (Chun and Jiang, 1998). The benefits of attending to spatial relations has also been observed in navigation studies with rodents, primates, children, and adults where use of contextual information facilitates search performance (Poucet et al., 1986; Hermer and Spelke, 1996; Gouteux et al., 2001; Gouteux and Spelke, 2001; Wang and Spelke, 2002). But the benefit of spatial structure on learning and memory is not exclusive to visual search and navigation tasks; it is also important for pedagogy (McCrink et al., 2014; McCrink and Galamba, 2015; McCrink and Shaki, 2016). Children better remember and utilize new information when it is presented to them in a culturally congruent spatial manner (i.e., left-to-right or right-to-left) (McCrink et al., 2014; McCrink and Galamba, 2015; McCrink and Shaki, 2016). English-speaking children perform better at locating a hidden object when presented with number labels increasing from left-to-right, the same direction in which English is read and written (Opfer et al., 2010). Conversely, Israeli children perform better when ordered information is presented from right-to-left, as in the Hebrew language (McCrink et al., 2014). Furthermore, observations of older children reveal that children, like adults, produce spatially dependent representations of temporal events in culturally congruent ways either from left-to-right or from right-to-left when instructed to present such information (Tversky et al., 1991). Thus, spatial attention seems to be a significant component of learning and memory throughout development, and is shaped by the broader context of the child.

Although studying spatial attention using explicit, one-dimensional tasks provides valuable insight into children’s spatial abilities for situations that are clearly spatial in nature, it is also important to investigate children’s attention to space in naturalistic environments. Children’s immediate, everyday environments have multiple layers of complexity, only one of which is space. Guiding children to focus on space in these naturalistic play environments bolsters their immediate performance on subsequent spatial tasks, like puzzle completion (Polinsky et al., 2017). Work on the independent production of spatial relations has found that, in spatial construction tasks, most adults choose to traverse through the same construction steps when recreating a block model, even though many different paths could result in construction of the correct replica (Cortesa et al., 2017). Importantly, it is this type of spatial skill (i.e., systematic block construction) early in childhood that is predictive of 3-year-old children’s early mathematical performance (Verdine et al., 2014a,b). In tasks like these, children and adults extract spatial information from a presented model and consider this information as they attempt to replicate the intended structure. Moreover, individual variability in spatial reasoning abilities across various dimensions of spatial cognition (e.g., Spatial Orientation, Mental Rotation, Spatial Visualization) is predictive of performance in STEM (Science, Technology, Engineering and Math) related disciplines like engineering (Hsi et al., 1997) and chemistry (see Wu and Shah, 2004 for a review). In fact, spatial ability measured in adolescence is predictive of individuals’ pursuit and completion of bachelor’s, master’s, and doctorate degrees in physical sciences, engineering, math, and computer science fields (Wai et al., 2009). Even early in life, observed individual variability in one spatial reasoning task is correlated with performance in another spatial reasoning task, even after controlling for verbal intelligence and age, as demonstrated in Möhring et al. (2015a,b) with 4- to 5-year-olds. Thus, the focus on spatial relations in more naturalistic tasks – and its positive relation to future math performance and performance in other non-math STEM disciplines – suggests that there is value in studying the development of attention to space.

Similar investigations of children’s propensity to focus on magnitudes and its relation to future mathematical performance can be seen in studies about spontaneous focus on numerosity (SFON). SFON serves as a measure of a child’s spontaneous tendency to focus attention on, and explicitly enumerate the exact number of, items in a set. A series of studies have concluded that SFON improves with age and is predictive of later numeracy and arithmetic skills and that this relation is reciprocal and long-lasting (Hannula and Lehtinen, 2005; Hannula et al., 2010; Hannula-Sormunen et al., 2015). Additionally, SFON is related to estimation skills; so it may function as a link between automatic processing of magnitudes and deliberate counting ability (Hannula et al., 2007). Moreover, guiding children to focus on number in a naturalistic environment boosts their subsequent SFON performance (Braham et al., 2018). Thus, SFON may serve as an implicit precursor that directs later interactions with environments containing mathematical elements. However, the development of children’s spontaneous focus on the spatial relations within their environment, and whether this capacity may serve as a precursor to performance on highly spatial mathematical and scientific concepts (e.g., proportions, geometry, engineering, chemistry etc.), remains unknown.

Because spatial concepts are deeply linked to numerical cognition (Mix et al., 2016), it is key to pursue a spatial equivalent of SFON. Specifically, the ability to reason about the *relation* between magnitudes has been linked to rational number reasoning skills, which are fundamental for understanding fractions and proportions (Möhring et al., 2015a,b; McMullen et al., 2016). Learning fractions is a notoriously difficult task for young children to learn; yet the innate, untrained processes that guide this thinking are present early in infancy and even in non-human primates (*Macaca mulatta*) and chicks (*Gallus gallus*) (Huttenlocher et al., 2002; McCrink and Wynn, 2007; Lortie-Forgues et al., 2015). For example, researchers were able to train rhesus macaques to determine the larger of two ratios, regardless of the absolute number of stimuli in each ratio (Drucker et al., 2016). In another study, 4-day-old chicks were trained to recognize certain proportions, and were able to recognize the target proportion even among novel ratios (Rugani et al., 2016). Despite the fact that children struggle to understand fractions represented with discrete amounts, if they are represented in continuous amounts children as young as 3 correctly reason about proportionality (Hurst and Cordes, 2018). In fact, even 6-month-old infants can reason about proportional relations between two continuous quantities when they are spatially represented (Duffy et al., 2005). Therefore, there is reason to believe that mathematically untrained children may be able to readily extract information regarding proportions when they are expressed non-symbolically, generating spatial magnitude information that the individual can elect to bring into focus.

Previous studies have aimed to measure the relation between spontaneous focus on spatial relations and formal fraction problem-solving (McMullen et al., 2011, 2013, 2014). In these studies, McMullen et al. (2011, 2013, 2014) measured children’s spontaneous focus on quantitative relations (SFOR), defined as “reasoning about the relationship, based on some quantifiable aspect(s), between two or more objects, sets, or symbols” (McMullen et al., 2014, p. 4). Children in these studies were presented a series of tasks that were designed to measure whether children would spontaneously focus on number, on quantitative relations, or on neither dimension when imitating the experimenter. For example, in one of the tasks children were introduced to two puppets who do “everything in exactly the same way,” meaning that whatever one dog did, so did the other. The researcher would go on to explain that the two dogs loved bread, then instructed children to watch carefully and then to do exactly as the researcher did. The challenge for the child was to identify along which dimension to focus on when deciding how much bread to feed the dog. For example, in one trial, the researcher had two halves of a felt cloth representing bread and “fed” one half to the puppet, whereas the child had “bread” that was cut into four fourths. If the child chose to give the puppet one of her pieces (one-fourth of the whole), then she was scored as exhibiting attention to numerosity because the child gave the same number of pieces of “bread” to the puppet as did the researcher (but not the same proportion of “bread”). Conversely, if the child gave the puppet two of her pieces (half of the whole), then she was scored as exhibiting attention to quantitative relations, meaning that she gave the same proportion of “bread” (but not the same number of “bread” pieces). Researchers found stable individual differences that predicted children’s later fraction performance, but not mathematical performance overall. Moreover, researchers found spontaneous focus on quantitative relations to be malleable and improvable through guided practice (McMullen et al., 2017). These findings suggest that children spontaneously focus on other magnitudes in addition to numerosity, and provide evidence of spatial cognition’s influence on mathematical performance. However, in these tasks, children were required to first reason about proportions, and subsequently choose *between* spatial extent and numerosity, even though these dimensions are usually positively related to each other.

Thus, to measure children’s spontaneous tendency to attend to absolute and relative spatial components of the environment (SFOS: Spontaneous Focus on Space), we provided children with spatially and mathematically rich scenes and asked them to recreate these scenes on their own after a brief delay. This task confers several benefits to the field of spatial and numerical cognition: (1) it requires low verbal and low symbolic ability, (2) linguistic fluency is not conflated with spatial attention, (3) we can independently measure children’s spontaneous focus on space and their spontaneous focus on number without pitting one against the another, and (4) it does not guide children to attend to the spatial aspects or the numerical aspects of the stimuli. Rather, children are only told to do just as the experimenter did, so it is truly spontaneous. As in SFON, the task used here (SFOS) does not require children to attend to the numerical relations between the objects with which they are asked to model the researcher. This overcomes the problem that previous work on SFOR encountered (McMullen et al., 2014), because here both the child and researcher are given the same objects with the same quantitative meanings. Thus, there is no implied aspect of the study that would indicate that the child should focus on proportions. This allowed us to simultaneously and independently measure the developmental trajectory of attention to space alongside attention to numeracy. Because we predicted that the trajectory of SFOS would be similar to that of SFON, children in the current study were evaluated for both their SFOS and SFON. Previous research has identified stable individual differences and rapid improvement in SFON during the preschool years, a trajectory that is reciprocally related to the counting abilities of children during this period, and is predictive of their future mathematical abilities (Hannula and Lehtinen, 2005). In the current paradigm, we predicted similar individual differences in children’s spontaneous focus on space and that this ability would rapidly improve throughout early childhood. Moreover, because SFOS encompasses aspects of both absolute and relative spatial information – information that facilitates young children’s reasoning about fractions (Jeong et al., 2007) – we predicted it would be positively correlated with proportional scaling ability (a domain linked to formal rational number knowledge; Möhring et al., 2015a,b).

In addition to developing a comprehensive measure of spontaneous focus on space, the naturalistic environment of the current study – a children’s museum – allowed us to evaluate the relations between parental spatial language input and children’s tendencies to spontaneously focus on spatial relations in their environment, as well as their performance on the highly spatial mathematical task of proportional reasoning. Previous research has provided evidence that spatial language heard by young children directs their attention to the spatial aspects of their environment (Gentner and Loewenstein, 2002; Gentner, 2003; Gentner et al., 2013). Relational language facilitates children’s understanding about spatial concepts, such as objects’ spatial relations to each other (Rattermann and Gentner, 1998). Words like *under* and *above* direct children to attend to aspects of their environment that may have gone unnoticed otherwise. Gentner and colleagues have found that spatial language may play a major role in the development of spatial-relational understanding, as language provides the tools with which one can think about and visualize space (Gentner and Loewenstein, 2002; Gentner, 2003; Casasola, 2005; Loewenstein and Gentner, 2005). Additionally, parents who use spatial language have children who do the same, and children’s spatial language is predictive of their performance on later spatial tasks (Pruden et al., 2011; Miller et al., 2017; Polinsky et al., 2017). The quality of children’s spatial language production (e.g., task-relevant or task-irrelevant) is predictive of their spatial skills above and beyond other demographic factors (Miller et al., 2017), and providing children with relevant verbal information prior to or during a spatial task significantly improves their ability to accurately recall spatial relations (Dessalegn and Landau, 2008, 2013; Miller et al., 2016). For these reasons, parental spatial language in the current study may be predictive of children’s SFOS and of their performance on a proportional reasoning task. Given previous work which has found that parents structure the world spatially for their child (McCrink and Shaki 2016), and that parent spatial language is linked to children’s early spatial abilities (Szechter and Liben, 2004; Loewenstein and Gentner, 2005; Pruden et al., 2011), we hypothesize that SFOS will be positively related to parental spatial language.

## Current Study

The current study evaluated the development of children’s spontaneous focus on space (SFOS), and how individual differences across this dimension may predict performance on highly spatial mathematical concepts like proportional reasoning. Previous studies of children’s SFON have found that individual differences in this dimension predict performance on numeracy and arithmetic; we therefore hypothesized that individual differences in the way children spontaneously attend to spatial relations within their environment may predict children’s subsequent performance on proportional reasoning. Moreover, we hypothesized that although children’s spontaneous focus on numerosity (SFON) has been found to significantly predict numeracy and arithmetic skills (Hannula and Lehtinen, 2005; Hannula et al., 2010; Hannula-Sormunen et al., 2015), children’s spontaneous focusing on space may be more predictive of their performance on mathematical concepts which are more spatial (e.g., fractions; Matthews et al., 2016; Lewis et al., 2016). Because a child’s early experiences with spatial language influences their spatial reasoning abilities (Gentner and Loewenstein, 2002; Gentner, 2003; Gentner et al., 2013) and because previous studies have found gender (Levine et al., 1999; Moore and Johnson, 2008; Pruden et al., 2011) and socioeconomic differences (Verdine et al., 2014a,b) play a role in the development of spatial concepts and skills, we also evaluated how parental and demographic factors (e.g., parental spatial language used during a spatial task, maternal education level, child’s gender/age/vocabulary) may uniquely contribute to children’s spontaneous focus on space and their proportional reasoning abilities. For these reasons, we examined here 3-to 6-year-old children’s performance on a proportional reasoning, SFOS, and SFON tasks [where their general ability to focus and recall non-quantitative information such as color was also measured (Spontaneous Focus on Color, or SFOC)] and parent language use during a dyadic building task. Finally, in our attempt to better represent the holistic experiences of children in our study, we asked parents to complete a vocabulary production questionnaire for their child (i.e., DVAP), and to indicate their child’s play preferences [engaging in a more (Legos^®^) or less (coloring) spatial task for free play].

## Materials and Methods

### Participants

Participants were 55 primarily English-speaking children aged three to six from a children’s museum or private kindergarten in New York City (*M*_age_ = 5;08-years-old, Range = 3;00 to 6;58). Parents of 24 children noted that their child had daily exposure and demonstrated some fluency in a language other than English (e.g., Russian, Mandarin, German, Spanish, French, Korean, Polish, Filipino, Punjabi, Hindi, Portuguese, Japanese, Creole). This sample included 30 male and 25 female participants. Participants were from a variety of socioeconomic backgrounds, but primarily clustered around middle- to high-SES, as gauged by parental education level (*M*_parentaleducationlevel_ = 4.47, range 1–5; where 1 corresponds to some high school education, 2 corresponds to high school graduate, 3 corresponds to some college education, 4 corresponds to college graduate, and 5 corresponds to post-baccalaureate education). This study was conducted in both informal and formal contexts, and our participants and their parents were relatively diverse (almost half of the children in our study spoke another language and the education level of parents in our study spanned the entire measurement scale). Written, informed consent was obtained from children’s parents prior to participation.

### Design

There were five parts to the current study: a toy preference task (in which parent–child dyads elected to either build with Lego^®^ pieces or to color with crayons before the tasks began), a Dyadic building task (consisting of Lego^®^ construction of a house), a joint Spontaneous Focus on Space and Spontaneous Focus on Numerosity task (SFOS and SFON; composed of two scene recreation tasks and two model imitation tasks), a Proportional Scaling task (modeled after Möhring et al., 2015a,b), and the Development Vocabulary Assessment for Parents (DVAP: Libertus et al., 2015). All children first played with their parent and then received the Dyadic Building task, then the SFOS and SFON tasks, and finally the Proportional Scaling task. The caregivers completed the DVAP while the children completed the SFOS, SFON, and Proportional Scaling tasks. A Canon FS300 camera was used to record each session for later coding.

#### Toy Preference Question

On the way to the experiment, parents were asked which activity – coloring, or playing with Lego^®^ pieces – their child would want to play with while the experimenter set up, and their response indicated. This measure was used to assess parents’ view of their children’s preferences for more-spatial or less-spatial activities.

#### Dyadic Building

This measure was used to evaluate the amount of type of parent spatial language used with their child during a building task.

##### Stimuli

**Figure 1:**
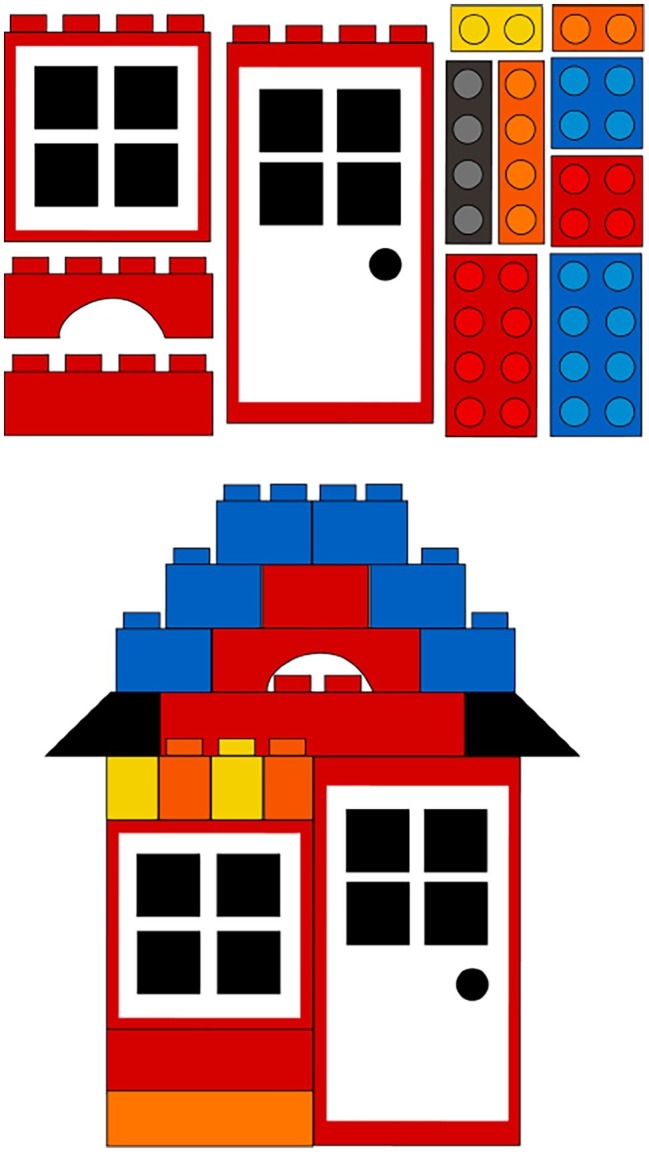
Dyadic building task materials and completed structure.

The building task involved the construction of a house made of 25 Lego^®^ pieces (Figure 1). Assembly of the Lego^®^ house required a large green Lego^®^ board, four orange blocks, seven red blocks, two yellow blocks, eight blue blocks, one red-and-white window pane piece, one red-and-white door piece, and an instruction manual containing pictures describing each step.

##### Procedure

For the building task, caregivers were instructed to work with their child to assemble the Lego^®^ figure using the provided instruction booklet (e.g., the experimenter stated, “Please work with your child to complete this building project,” to emphasize the interactive nature of the task). This measure ended when the caregiver indicated they were finished and the figure was completely assembled. Children were given a sticker after completing this task.

##### Coding

Parent spatial input during this task was coded using the spatial language manual developed by Cannon et al. (2007). Parental spatial input was composed of primarily spatial language (including the following categories of words: Spatial Dimensions, Shape Terms, Location and Direction, Continuous Amount, Deictics, Spatial Features and Properties, and Pattern), with an additional measure of spatial gesture. Specifically, we were most interested in parents cueing children to the relations between entities on the instructional card (e.g., the model of what was to be built) and the current state of the pieces in front of the child that they were working on. This is known as Relational Gestures, wherein a caregiver touched both material and instructional components of the spatial activity with the purpose of comparing the two (Goldin-Meadow et al., 1999; Alibali and Nathan, 2007, 2012). Every time parents touched the building pieces and the related instructional components to compare the two they received one point, and the sum of all these instances became their gesture score. Because this task was untimed, and because some parents talked more or less than others, parents received a spatial language production score (e.g., percentage) that was calculated by taking total spatial words produced and dividing that sum by total words used. All parents either used spatial language, relational gesture, or a combination of both during this dyadic building task.

#### Spontaneous Focus on Space Task

Four items were used to measure the children’s SFOS: two scene recreation trials and two model imitation trials (see Hannula and Lehtinen, 2005). In total, the SFOS task took on average 6–8 min. As a measure of Spontaneous Focus on Numerosity, children’s SFON was measured. As a measure of general memory for non-quantitative dimensions of the scene, children’s Spontaneous Focus on Color was measured (e.g., their attention to a recall of the color of the entities used in the recreation and imitation tasks). Across these three dimensions, children received scores for each of the four different trials (two scene recreation trials and two model imitation trials). Scores were then summed for each dimension resulting in a composite SFOS score, SFON score, and SFOC score. For ease of comparability between dimensions, all individual dimension raw scores were changed to percentages out of total points possible for that construct (see Table 1).

**Table 1:** Descriptive Information for dependent measures.

	Min	Max	*N*	Mean	Std. Dev.
Proportional scaling error distance SFOC SFON SFOS	14.00 0 0 4.25	113.10 100 100 94.50	55 55 55 55	49.42 55.30 58.75 54.95	23.67 30.53 32.49 21.91

*For consistency, the maximum points possible on all the spontaneous imitation measures was 100, where children’s raw scores were converted to percentages out of total points possible for each metric*.

##### Scene Recreation Trials

###### Stimuli

**Figure 2:**
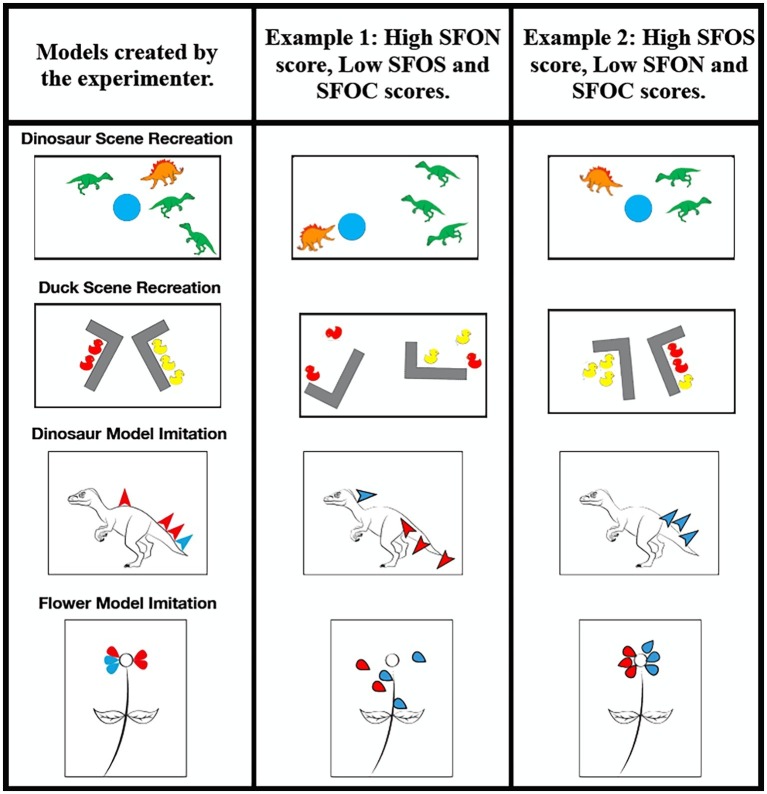
Duck Scene Recreation trial and Dinosaur Scene Recreation trial. The dimensions of the white board were 51.6 cm × 29.0 cm. Dinosaur and Flower Stamp Modeling Task. The above images were printed on 8.5 × 11.0 in paper. For both types of tasks the experimenter modeled a version in front of the child (left column) and then gave the child the materials and asked the child to do just as the they had done. Examples of how children could have performed across the dimensions of SFON (center column), SFOS (rightmost column) and SFOC (general memory performance) are depicted.

Both the dinosaur scene recreation and duck scene recreation trials were conducted on a rectangular white board 51.6 cm by 29.0 cm with a black border. In the dinosaur scene recreation trial, three green Tyrannosaurus rex toys, one brown Stegosaurus toy and a blue foam circle representing a watering hole were used (Figure 2). Distractor items comprising two extra green Tyrannosaurus rex toys, one orange Velociraptor toy, one orange Spinosaurus toy, and one orange/dark brown Triceratops toy were provided. Five distractor items for the dinosaur task were chosen so that children had double the entities to choose from when recreating the scene they were just shown. Because performance on SFON and SFOS could be due to more general attention or memory differences throughout development, we planned to use Spontaneous Focus on Color as a measure of general recall ability (SFOC). Thus, we aimed to provide the children with three distractor dinosaurs that were brown/orange in hue so that they could incorrectly choose to make the majority color of dinosaurs on the board orange and not green. That is, if children noticed that three out of the four dinosaurs were similarly colored in the scene, then when they were subsequently given the jumbled box of dinosaurs they could have incorrectly recalled that three of the dinosaurs on the board were orange/brown and one was green. Half of the children witnessed the experimenter use three orange/brown dinosaurs and one green dinosaur to create the scene whereas the other half witnessed the experimenter use three green dinosaurs and one orange/brown dinosaur. Materials for the duck scene recreation trial were two yellow L-shaped walls made from Duplo blocks and a total of five rubber ducks (Figure 2). The five rubber ducks were either red or blue in color. In a given session, three ducks were of one color, and two ducks were of the other color. In total, there were 10 distractor items, six of which were purple ducks. The remaining distractor items varied depending on the condition. If three red ducks and two blue ducks were used in the trial, the distractor items included three red ducks and one blue duck. If two red ducks and three blue ducks were used in the trial, the distractor items included four red ducks.

###### Procedure

After completing the Dyadic Building Task with their caregiver, participants began the two scene recreation trials. The setup of each scene was prefaced with, “Watch carefully what I do, and then you do it just like I did.” The experimenter then laid out the scene for the child (exact layouts, with dimensions, are presented in Figure 2). Once the scenes were set up, participants were encouraged to look carefully at the scene. Then, all objects were removed from the white board, and placed into a bowl. This bowl contained distractor objects of similar and distinct colors (again to aid in the measurement of SFOC). The participant was then encouraged to recreate each scene, and tell the experimenter when they were done. The sequence in which the scene recreation trials were presented varied to eliminate order effects. After completing the scene recreation trials, children were rewarded with a sticker.

###### Coding

For this coding scheme, as well as the coding scheme for the stamp modeling trial, we sought to determine which aspects a child focused on when encountering a novel activity. These aspects included absolute spatial relations, relative spatial relations, number, and color. Absolute spatial relations refer to the location placement of objects or stamps on certain parts of the scene or picture. Relative spatial relations involve the placement and orientation of objects or stamps relative to each other. For example, if the target angle between two objects was 90°, children received accurate scores if the object they oriented was angled more than 70° but less than 110°. Number metrics capture which quantitative aspects of the trials children focused on (e.g., accuracy in “how many”). Finally, a color accuracy measure captured whether or not children focused on the specific colors used during the trials, as a measure of general memory performance (SFOC). Importantly, to accommodate variability in partially correct answers, partial points were systematically granted within each of the coding metrics. Specifically, because across all the recreation and imitation tasks there were grounding entities that were less numerous than others (e.g., one watering hole vs. four dinosaurs or two L-shaped blocks vs. six ducks), lack of spontaneously focusing on the spatial relation of these grounding entities on the board was considered more egregious and coded as a larger error than incorrectly placing one of the more numerous entities.

##### Dinosaur Scene Recreation Trial

###### Absolute Metrics

The Watering Hole metric referred to the placement of the watering hole in the center of the board. Participants received no points if the watering hole was not located on the center of the board. Participants received a score of 1 point if the watering hole was put in the center of the board.

The Dinosaurs Experimenter Used metric pertained to the placement of any four dinosaurs in the four correct locations on the board. Participants received no points if no dinosaurs were positioned in any of the four correct locations and received partial credits corresponding to how many correct dinosaur placements were made (i.e., out of four possible correct placements). Participants received a score of 0.25 points if one dinosaur was placed in one of the correct locations on the board. Participants received a score of 0.50 points if two dinosaurs were set in two of the correct locations on the board. Participants received a score of 0.75 points if three dinosaurs were placed in three of the correct locations on the board. Participants received a score of 1 point if four dinosaurs were arranged in all four of the correct locations on the board.

The Oddball Dinosaur metric related to the placement of the dinosaur not facing the watering hole. Participants received no points if all dinosaurs were facing the watering hole. Participants received a score of 1 point if there was one dinosaur not facing the watering hole. Importantly, children could have chosen any dinosaur on the board to face away from the watering hole and receive 1 point.

###### Relative Metrics

The Orientation of Dinosaurs metric referred to the alignment of the dinosaurs with respect to each other. The lone green dinosaur on one side of the board must have been positioned to make a 90° angle with the brown dinosaur on the other side of the board. The second green dinosaur, located below the brown dinosaur, must have been placed to be parallel to the length of the board. The third green dinosaur, located below the second green dinosaur, must have been set to face the same direction and in a straight line as the lone green dinosaur on the opposite side. Participants received no points if none of the dinosaurs on the board were oriented correctly and received partial credits corresponding to how many correct dinosaur placements were made (i.e., out of four possible correct orientation placements). Participants received a score of 0.25 points if one dinosaur was oriented correctly. Participants received a score of 0.50 points if two dinosaurs were oriented correctly. Participants received a score of 0.75 points if three dinosaurs were oriented correctly. Participants received a score of 1 point if four dinosaurs were oriented correctly.

The Orientation of the Oddball Dinosaur metric concerned the alignment of the second green dinosaur opposite the watering hole. Participants received 1 point if the dinosaur that was not facing the watering hole was facing opposite the watering hole (e.g., its entire body facing away from the watering hold). Participants received no points if the dinosaur was not oriented completely opposite the watering hole (e.g., if it was only 90–120° oriented away from the watering hole as compared to 180°). For example, if children chose the third green dinosaur located at the bottom right of the board to make the Oddball Dinosaur, they would receive 0.25 points for placing one of four dinosaurs in the correct portion of the board, no partial points for Relative Metrics since this rotated dinosaur would not be correctly oriented relative to the other dinosaurs if it were facing away from the watering hole, and 1 point for the Oddball Dinosaur category, for making one dinosaur face away from the watering hole and 1 point for the Orientation of the Oddball Dinosaur for orienting it facing completely opposite of the watering hole.

###### Number Metrics

The Exact Number of Dinosaurs Used metric pertained to total quantity of dinosaurs on the board. Participants received no points if more or less than four dinosaurs were placed on the board. Participants received a score of 1 point if exactly four dinosaurs were set on the board.

Number of Dinosaurs on Each Side metric related to the quantity of dinosaurs on either side of the board. Participants received no points if there were neither three dinosaurs on one side of the board nor one dinosaur on one side of the board. Participants received a score of 0.50 points if three dinosaurs were positioned on one side of the board and more or less than one dinosaur were arranged on the other side of the board. Participants received a score of 0.50 points if one dinosaur was put on one side of the board and more or less than three dinosaurs were set on the other side of the board. Participants received a score of 1 point if there were three dinosaurs on one side of the board and one dinosaur on the other side of the board. Notice, this is different than the absolute metrics for the Dinosaurs the Experimenter used metric, because children could have placed the correct number of dinosaurs on the wrong side of the board here (or could have even chosen to segment the board into a top and bottom half instead of left and right) and still received full points, whereas the absolute metrics for location of the dinosaurs concerns the absolute locations of where on the board dinosaurs are placed.

###### Color Metrics

Color Grouping of Dinosaurs metric concerned the placement of dinosaurs of a single color on one side of the board and the placement of dinosaurs of mixed colors on the other side of the board. Participants received no points if dinosaurs were not arranged on two, distinct, left/right sides of the board from which to determine separate color groups. Participants received a score of 0.50 if one or more dinosaurs of a single color were placed on either side of the board. Participants received a score of 0.50 if one or more dinosaurs of mixed color were put on either side of the board. Participants received a score of 1 point if one or more dinosaurs of a single color were set on one side of the board and dinosaurs of mixed color were arranged on the other side of the board.

Color of Oddball Dinosaur metric referred to the color of the dinosaur facing away from the watering hole. Participants received no points if the dinosaur facing away from the watering hole was not the correct color based on what they saw the experimenter use. Participants received a score of 1 point if the dinosaur facing away from the watering hole was the same color as the one the experimenter used (see Figure 2 for examples).

##### Duck Scene Recreation Trial

###### Absolute Metrics

The Angles metric related to the placement of the two, L-shaped Duplo blocks in the center of the board. Participants received no points if the blocks were not placed on the center of the board. Participants received a score of 0.50 points if only one block was set in the center of the board. Participants received a score of 1 point if both blocks were put in the center of the board.

The Ducks Experimenter Used metric pertained to the placement of any five ducks in the five correct locations on the board. Participants received no points if no ducks were placed in any of the five correct locations and received partial credits corresponding to how many correct duck placements were made (i.e., out of five possible correct placements). Participants received a score of 0.20 points if one duck was put in one of the correct locations on the board. Participants received a score of 0.40 points if two ducks were arranged in two of the correct locations on the board. Participants received a score of 0.60 points if three ducks were positioned in three of the correct locations on the board. Participants received a score of 0.80 points if four ducks were put in four of the correct locations on the board. Participants received a score of 1 point if five ducks were placed in all five of the correct locations on the board.

The Oddball Duck metric concerned the placement of a duck not facing the L-shaped Duplo block to which it was closest. Participants received no points if all ducks were facing the block. Participants received a score of 1 point if there was one duck not facing the L-shaped block the block.

###### Relative Metrics

The Orientation of Angles metric referred to the alignment of the L-shaped Duplo blocks with respect to the participant. Participants received no points if the long ends of both blocks were positioned away from the participant. Participants received a score of 0.50 points if the short end of only one block was set away from the participant. Participants received a score of 1 point if the short ends of both blocks were put away from the participant.

The Correctness of Angle Degrees metric related to the orientation of the L-shaped Duplo blocks with respect to each other. Participants received 0 points if neither of the vertices of both blocks were pointed toward the midline of the board. Participants received 0.50 points if only one of the vertices of a block was pointed toward the midline of the board. Participants received 1 point if the vertices of both blocks were pointed toward the midline of the board.

The Orientation of Non-Oddball Ducks metric concerned the direction four ducks faced relative to the L-shaped blocks. Participants received no points if no ducks were put to face the L-shaped block to which the ducks are closest and received partial credits corresponding to how many correct duck placements were made (i.e., out of four possible correct orientation placements). Participants received a score of 0.25 points if one duck was placed to face the L-shaped block to which the duck was closest. Participants received a score of 0.50 points if two ducks were set to face the L-shaped blocks to which the ducks were closest. Participants received a score of 0.75 points if three ducks were placed to face the L-shaped blocks to which the ducks were closest. Participants received a score of 1 point if four ducks were positioned to face the L-shaped blocks to which the ducks were closest.

The Orientation of the Oddball Duck metric related to the alignment of a duck which faces opposite the L-shaped block to which the oddball duck was closest. Participants received a score of 1 if one duck was facing directly opposite the L-shaped block to which the oddball duck was closest. Participants received no points if the duck was not oriented completely opposite the L-shaped block it was closest to (e.g., if it was only 90–120° oriented away from the L-shaped block as compared to 180°).

###### Number Metrics

The Exact Number of Ducks Used metric pertained to total quantity of ducks on the board. Participants received no points if more or less than five ducks were arranged on the board. Participants received a score of 1 point if exactly five ducks were set on the board.

The Number of Ducks on Each Side metric referred to the quantity of ducks on either side of the board. Participants received no points if there were neither three ducks on one side of the board nor two ducks on one side of the board. Participants received a score of 0.50 points if three ducks were positioned on one side of the board and more or less than two ducks were placed on the other side of the board. Participants received a score of 0.50 points if two ducks were put on one side of the board and more or less than three ducks were set on the other side of the board. Participants received a score of 1 point if there were ducks on one side of the board and two ducks on the other side of the board. Notice, this is different than the absolute metrics for the Ducks the Experimenter used metric, because children could have placed the correct number of ducks on the wrong side of the board here (or could have even chosen to segment the board into a top and bottom half instead of left and right) and still received full points, whereas the absolute metrics for location of the ducks concerns the absolute locations of where on the board ducks are placed.

###### Color Metrics

The Color Grouping of Ducks metric pertained to the placement of ducks of one color on one side of the board, and the placement of ducks of both the same color and a second color on the other side of the board. Participants received no points if ducks were not arranged on two, distinct, left/right sides of the board from which to determine separate color groups. Participants received a score of 0.50 if one or more ducks of a single color were located on either side of the board. Participants received a score of 0.50 if one or more ducks of mixed color were fixed on either side of the board. Participants received a score of 1 point if one or more ducks of a single color were placed on one side of the board and ducks of mixed color were put on the other side of the board.

The Color of Oddball Duck metric related to the color of the duck facing away from the L-shaped block to which the oddball duck was closest. Participants received no points if the duck facing away from the L-shaped block to which it was closest was not of the same color as the oddball duck used by the experimenter. Participants received a score of 1 point if the duck facing away was of the same color as the oddball duck used by the experimenter.

##### Model Imitation Trials

Both the dinosaur and flower stamping trials utilized the same red stamp pad and blue stamp pad. They were adapted from a similar technique to measure SFON by Hannula and Lehtinen (2005).

###### Stimuli

For the dinosaur stamping trial, one copy of a picture depicting a left facing dinosaur without scales on its back for the experimenter and another identical copy for the participant and a stamp made in the shape of an arrow head were required. For the flower stamping trial, one copy of a picture depicting a flower without petals for the experimenter and another identical copy for the participant and a stamp made in the shape of a teardrop were required. See Figure 2 for a schematic of these models as well as stamp placement.

###### Procedure

The procedure for the stamp replication was similar to the procedure outlined for the scene recreation trials. The stamping activity was prefaced with “Watch carefully what I do, and then you do it just like I did.” After the experimenter stamped on a picture (i.e., dinosaur and flower), participants stamped on a matching picture with the additional ability to reference the experimenter’s stamped picture. During the dinosaur stamping trial, four arrowhead-shaped scales were stamped onto a dinosaur picture using red and blue stamping ink. A trio of red and blue scales were stamped on one end of the dinosaur, and one scale of a single color was stamped on another part of the dinosaur. During the flower stamping trial, five teardrop-shaped petals were stamped onto a picture of a flower without petals. In either red or blue ink, three petals of mixed color were stamped on one side of the flower and two petals of the same color were stamped on the other side of the flower. The order of the stamping trials was counterbalanced. Children were given a sticker upon completing both stamping trials.

###### Coding

Dinosaur Stamping Trial – Absolute metrics. The Scale Placement metric referred to the number of arrowhead-shaped stamps on the participant’s dinosaur that were stamped in the same four locations as the experimenter’s stamped dinosaur. Participants received no points if no stamps were set in any of the four correct locations on the picture and received partial credits corresponding to how many correct stamp placements were made (i.e., out of four possible correct stamp placements). Participants received a score of 0.25 points if one stamp was put in one of the correct locations on the picture. Participants received a score of 0.50 points if two stamps were positioned in two of the correct locations on the picture. Participants received a score of 0.75 points if three stamps were set in three of the correct locations on the picture. Participants received a score of 1 point if four stamps were placed in all four of the correct locations on the picture.

The Scale Angle metric referred to the tilt of the arrowhead-shaped stamp in relation to the back of the dinosaur on the page. Participants received no points if no stamps were pointed in the correct direction in relation to the dinosaur’s back and received partial credits corresponding to how many correctly angled stamps were made (i.e., out of four possible correct angles). Participants received a score of 0.25 points if one stamp was pointed in the correct direction in relation to the dinosaur’s back. Participants received a score of 0.50 points if two stamps were pointed in the correct direction in relation to the dinosaur’s back. Participants received a score of 0.75 points if three stamps were pointed in the correct direction in relation to the dinosaur’s back. Participants received a score of 1 point if four stamps were pointed in the correct direction in relation to the dinosaur’s back.

###### Relative Metrics

The Scale Groups metric related to the placement of stamps in two, distinct, left/right groups on the page. Participants received no points if stamps were not arranged in two, distinct groups. Participants received a score of 1 point if stamps were arranged in two, distinct groups.

The Scale Orientation metric referred to the alignment of stamps with respect to each other. Participants received a score of 0.25 point if one stamp was placed with the correct scale angle. Participants received a score of 0.50 points if two stamps were arranged with the correct scale angles. Participants received a score of 0.75 points if three stamps were put with the correct scale angles. Participants received a score of 1 point if four stamps were placed with the correct scale angles.

###### Number Metrics

The Exact Number of Scale Stamps Used metric pertained to total quantity of scale stamps on the page. Participants received no points if more or less than four stamps were put on the page. Participants received a score of 1 point if exactly four stamps were placed on the page.

The Number of Scale Stamps on Each Side metric related to the quantity of stamps on either side of the page. Participants received no points if there were neither three stamps on one side of the page nor one stamp on one side of the page. Participants received a score of 0.50 points if three stamps were positioned on one side of the page and more or less than one stamp were put on the other side of the page. Participants received a score of 0.50 points if one stamp was placed on one side of the page and more or less than three stamps were set on the other side of the page. Participants received a score of 1 point of there were three stamps on one side of the page and one stamp on the other side of the page. Again, this is different than the Absolute Scale Placement metric, because children could have segmented the sides of the paper as top and bottom and still receive points here as long as they segmented the quantity of stamps on either side of the page.

###### Color Metrics

The Color Grouping of Scale Stamps metric referred to the placement of stamps of a single color on one side of the page and the placement of stamps of mixed colors on the other side of the page. Participants received no points if stamps were not arranged on two, distinct sides of the page from which to determine separate color groups. Participants received a score of 0.50 if one or more stamps of a single color were placed on either side of the page. Participants received a score of 0.50 if one or more stamps of mixed color were fixed on either side of the page. Participants received a score of 1 point if one or more stamps of a single color were put on one side of the page and stamps of mixed color were set on the other side of the page.

##### Flower Stamping Trial

###### Absolute Metrics

The Petal Placement metric referred to the number of teardrop-shaped stamps on the participant’s flower that were stamped in the same four locations as the experimenter’s stamped flower. Participants received a score of 0.20 points if one stamp was arranged in one of the correct locations on the picture. Participants received a score of 0.40 points if two stamps were positioned in two of the correct locations on the picture. Participants received a score of 0.60 points if three stamps were put in three of the correct locations on the picture. Participants received a score of 0.80 points if four stamps were placed in four of the correct locations on the picture. Participants received a score of 1 point if five stamps were set in all five of the correct locations on the picture.

The Petal Angle metric referred to the direction in which the vertex of the teardrop-shaped stamp was pointing. Participants received no points if no stamps were pointed toward the center of the flower petal and received partial credits corresponding to how many correctly angled stamps were made (i.e., out of five possible correct angles). Participants received a score of 0.20 points if one stamp was pointed toward the center of the flower petal. Participants received a score of 0.40 points if two stamps were pointed toward the center of the flower petal. Participants received a score of 0.60 points if three stamps were pointed toward the center of the flower petal. Participants received a score of 0.80 points if four stamps were pointed toward the center of the flower petal. Participants received a score of 1 point if four stamps were pointed toward the center of the flower petal.

###### Relative Metrics

The Petal Groups metric pertained to the placement of stamps in two, distinct, left/right groups on the page. Participants received no points if stamps were not arranged in two, distinct groups. Participants received a score of 1 point if stamps were arranged in two, distinct groups. Notice, this is different than the Absolute Petal Placement metric because the child could have made one group of stamps far away from the center of the flower along the border of the paper and the other stamps far away from the center of the flower along the other boarder of the paper and received points here for this metric whereas for the Absolute Petal Placement metric they needed to place the stamps in the correct location of the paper.

The Petal Orientation metric related to the alignment of stamps with respect to each other. Participants received no points if no stamps were placed with the correct petal angle, relative to each other. Participants received a score of 0.20 points if one stamp was positioned with the correct petal angle. Participants received a score of 0.40 points if two stamps were arranged with the correct petal angles. Participants received a score of 0.60 points if three stamps were set with the correct petal angles. Participants received a score of 0.80 points if four stamps were put with the correct petal angles. Participants received a score of 1 point if five stamps were placed in the correct locations and with the correct petal angles. Notice, children could have correctly oriented the petals relative to each other anywhere on the page and received points for this metric; they did not need to place them in the correct location or use the correct number of stamps or use the correct color of stamps to correctly orient petals relative to each other the way that the experimenter had demonstrated.

###### Number Metrics

The Exact Number of Petal Stamps Used metric referred to total quantity of petal stamps on the page. Participants received no points if more or less than five stamps were put on the page. Participants received a score of 1 point if exactly five stamps were set on the page.

The Number of Petal Stamps on Each Side metric pertained to the quantity of stamps on either side of the page. Participants received no points if there were neither three stamps on one side of the page nor two stamps on one side of the page. Participants received a score of 0.50 points if three stamps were placed on one side of the page and more or less than two stamps were arranged on the other side of the page. Participants received a score of 0.50 points if two stamps were positioned on one side of the page and more or less than three stamps were set on the other side of the page. Participants received a score of 1 point if there were three stamps on one side of the page and two stamps on the other side of the page. Like described above, this is different than the Absolute Petal Placement metric, because children could have segmented the sides of the paper as top and bottom and still receive points here as long as they segmented the quantity of stamps on either side of the page.

###### Color Metrics

The Color Grouping of Petal Stamps metric referred to the placement of stamps of a single color on one side of the page and the placement of stamps of mixed colors on the other side of the page. Participants received no points if stamps were not arranged on two, distinct sides of the page from which to determine separate color groups. Participants received a score of 0.50 if one or more stamps of a single color were located on either side of the page. Participants received a score of 0.50 if one or more stamps of mixed color were arranged on either side of the page. Participants received a score of 1 point if one or more stamps of a single color were situated on one side of the page and stamps of mixed color were placed on the other side of the page.

#### Proportional Scaling Task

##### Stimuli

The proportional scaling task, adapted from Möhring et al. (2015a,b), was composed of 16 pictures. On each picture, a red and blue rectangle, representing cherry juice and water, respectively, were vertically stacked on top of each other. Each rectangle was 2 cm wide and its height varied based on a systematic design. A horizontal 12-cm black line below each rectangle served as a scale. A single cherry printed next to the left end of the scale indicated faint sweetness. A heap of cherries printed next to the right end of the scale indicated strong sweetness. Each page contained a different combination of cherry juice and water. The red rectangle representing cherry juice varied in four parts, (1.5, 2, 2.5, or 3 cm), and the total amount varied in four parts (3, 6, 9, or 12 cm), yielding 16 unique combinations in which the scaling ranged from large (a 4-fold difference between the picture and scale) to small (a 1:1 ratio between the picture and scale). In addition to the pictures developed by Möhring et al. (2015a,b), three novel combinations of cherry juice and water were made and used for practice trials with feedback of the proportional scaling task.

##### Procedure

Participants were asked to gauge the sweetness of a juice-water mixture and generalize the proportion to a rotated scale. Researchers prefaced the task by saying, “This is my friend, Wally, the bear. Wally likes to make his own cherry juice by mixing cherry juice and water. Wally has made a lot of juice today and it’s our job to tell him how sweet the juice is.”

The first three trials were practice trials in which the sweetness of a glass of cherry juice was determined by participants pointing to part of the rotated scale and given corrective feedback if necessary. The first practice trial depicted a large glass of juice with very little water and mostly cherry juice. The researcher first pointed to the red bar and said, “This is how much cherry juice Wally has put in the cup.” The researcher then pointed to the blue bar and said, “This is how much water Wally has put in his cup.” Researchers pointed to the scale line located below the cup of juice and said, “How sweet do you think this cup of juice is? The single cherry means we think it is not sweet at all. The heap of cherries means we think it is very sweet. I’m going to show you how sweet this juice is.” The researcher then pointed to the correct location on the scale, which was closer to the heap of cherries, indicating high sweetness.

In the second practice trial, children were shown a picture of a glass with mostly water and a very little cherry juice and told, “Now look at this cup of juice. Can you show me how sweet this juice is by touching the line? Remember, the closer to the single cherry means it is not sweet at all and the closer to the heap of cherries means the sweeter the juice is.” The children were allowed to point at the scale, and given corrective feedback if necessary. For the third practice trial, children were shown a picture of a cup of juice with mostly cherry juice and some water and given the same instructions as in the second trial. Participants finished the remaining 16 trials without experimenter intervention. Once the task was completed, children were given a sticker.

##### Coding

The error distances between the correct proportional distance and the participants’ response were measured for all 16 trials. For example, a participant who saw a standard corresponding to 2 of 3 cm, and marked a location corresponding to 8 of 12 cm on the scale, would have 0 error distance. A participant who saw this same standard, but marked the location corresponding to 10 of 12 cm, would have a 2-cm discrepancy between the marked and correct answer.

#### Developmental Vocabulary Assessment for Parents

The DVAP (Libertus et al., 2015) is a questionnaire consisting of 212 words. Nouns, verbs, and adjectives of increasing difficulty (e.g., puppy and sternum, jumping and incarcerating, red and incandescent, from easiest to hardest, respectively) are included in the questionnaire. This questionnaire was completed by caregivers while their children participated in the SFOS, SFON, and proportional scaling tasks. The subjects’ caregivers were asked to select each word they have heard spoken by their child. Caregivers were also asked to indicate the proportion of time their child was exposed to languages other than English (if applicable) and whether the person completing the questionnaire was the child’s primary caregiver. The score for each child’s DVAP questionnaire is the sum of the number of words circled.

## Results

### Measuring Children’s Spontaneous Focus on Space

#### Developmental Trajectory of Spontaneous Focus on Space

To address whether a developmental trajectory for Spontaneous Focus of Space exists, and to address its relatability to that of Spontaneous Focus on Numerosity, we first conducted a regression with *z*-scored SFOS as the dependent variable and age (as a continuous variable), gender (female or male), and general memory performance across the imitation tasks (*z*-scored SFOC score) as independent variables. (Note that due to different base rate attainments for SFON, SFOS, and SFOC, their values were converted to z-scores). Together, these factors significantly predicted SFOS performance, *F*(3, 51) = 48.1, *p* < 0.001, ∆*R*^2^ = 0.74 (Table 2). Within this model, child’s age at test was a significant predictor of SFOS performance above and beyond general memory performance, *β*_age_ = 0.22, SE = 0.08, *t*(51) = 2.65, *p* = 0.01. Spontaneous Focus on Color (SFOC) also significantly predicted SFOS score, *β*_SFOC_ = 0.74, SE = 0.08, *t*(51) = 9.02, *p* < 0.001. However, gender was not a significant predictor of SFOS performance, *β*_gender_ = −0.03, SE = 0.14, *t*(51) = −0.38, *p* = 0.72. We next conducted a similar regression with *z*-scored SFON as the dependent variable and found a significant relation, *F*(3, 51) = 70.46, *p* < 0.001, ∆*R*^2^ = 0.81 between age, gender, SFOC, and SFON performance (Table 3). Within this model, child’s age at test was a significant predictor of SFON performance again above and beyond Spontaneous Focus on Color (general memory performance), *β*_age_ = 0.16, SE = 0.07, *t*(51) = 2.3, *p* = 0.03. SFOC significantly predicted SFON score, *β*_SFOC_ = 0.81, SE = 0.07, *t*(51) = 11.53, *p* < 0.001. However, gender was not a significant predictor of SFON performance, *β*_gender_ = −0.09, SE = 0.12, *t*(51) = −1.46, *p* = 0.15. These findings suggest an increased tendency to spontaneously focus on space and spontaneously focus on numerosity, although heavily dependent on a child’s ability to recall a scene (SFOC), with maturation.

**Table 2:** Summary of linear regression analysis for three variables (child’s age at test, gender, *z*-scored SFOC) predicting Spontaneous Focus on Space.

Variable	*B*	SE *B*	*β*
Constant Age at test *Z*-scored SFOC Gender Adjusted *R*^2^ *F*	−0.99 0.20 0.74 −0.05 0.74 48.1^**^	0.39 0.08 0.08 0.14	0.22* 0.74** −0.03	

*B, unstandardized regression coefficient; SE, standard error; *β*, standardized regression coefficien*t*; ∆*R*^2^, difference in the proportion of variance explained*.

* *p < 0.05*,

** *p < 0.01*.

**Table 3:** Summary of linear regression analysis for three variables (child’s age at test, gender, *z*-scored SFOC) predicting Spontaneous Focus on Numerosity.

Variable	*B*	SE *B*	*β*
Constant Age at test *Z*-scored SFOC Gender Adjusted *R*^2^ *F*	−0.67 0.15 0.81 −0.18 0.81 70.46^**^	0.34 0.07 0.07 0.12	0.16^*^ 0.81^**^ −0.09	

*B, unstandardized regression coefficient; SE, standard error; *β*, standardized regression coefficient; ∆*R*^2^, difference in the proportion of variance explained*.

* *p < 0.05*,

** *p < 0.01*.

#### Parent/Family Factors and Spontaneous Focus on Space

Next, we evaluated whether other factors like the parental education level, parent’s use of spatial language, or the free-play option parents and children chose (a Lego building task or a coloring task) to complete influenced children’s SFOS and/or SFON performance above and beyond child’s age and SFOC performance (general memory performance). A linear regression was conducted with *z*-scored SFOS as the dependent variable, and age, child’s gender, *z*-scored SFOC, parental education level, parent’s use of spatial language and the free-play option as independent variables. The overall model significantly predicted children’s spontaneous focus on space, *F*(6, 24) = 13.7, *p* < 0.001, ∆*R*^2^ = 0.72 (Table 4). The addition of gender [*β*_childgender_ = −0.06, SE = 0.19, *t*(24) = −0.59, *p* = 0.56], parental education level [*β*_parentaleducationlevel_ = −0.03, SE = 0.12, *t*(24) = −0.23, *p* = 0.82], parent’s use of spatial language [*β*_parentspatiallanguage_ = −0.17, SE = 2.97, *t*(24) = −1.54, *p* = 0.14], and free-play option [*β*_freeplayoption_ = 0.10, SE = 0.19, *t*(24) = 0.85, *p* = 0.41] did not explain any additional variance. In this model, only age, *β*_age_ = 0.26, SE = 0.09, *t*(24) = 2.2, *p* = 0.04, and SFOC (general memory performance), *β*_SFOC_ = 0.66, SE = 0.11, *t*(24) = 5.93, *p* < 0.001, predicted SFOS performance. To evaluate whether these additional factors better predicted SFON performance, a linear regression was conducted with SFON scores as the dependent variable, and age, *z*-scored SFOC, parental education level, parent’s use of spatial language, and free-play option as independent variables. The overall model significantly predicted children’s spontaneous focus on numerosity, *F*(6, 24) = 13.84, *p* < 0.001, ∆*R*^2^ = 0.72 (Table 5). The addition of child’s age, [*β*_age_ = 0.07, SE = 0.10, *t*(24) = 0.55, *p* = 0.59], gender [*β*_childgender_ = −0.13, SE = 0.20, *t*(24) = −1.16, *p* = 0.26], parental education level [*β*_parentaleducationlevel_ = −0.02, SE = 0.13, *t*(24) = −0.15, *p* = 0.88], parent’s use of spatial language [*β*_parentspatiallanguage_ = −0.18, SE = 3.15, *t*(24) = −1.64, *p* = 0.11], and free-play option [*β*_freeplayoption_ = 0.06, SE = 0.21, *t*(24) = 0.49, *p* = 0.63] did not explain any additional variance. In this model, only SFOC (general memory performance), *β*_SFOC_ = 0.78, SE = 0.12, *t*(24) = 6.99, *p* < 0.001, predicted SFON performance. These findings reveal no significant relation between parental factors like parental education level, parent’s use of spatial language, or free-play activity choice and children’s SFOS and SFON performance. They do reiterate age-related effects of spontaneously focusing on space and predictive relations between general memory performance (SFOC) and SFOS and SFON performance.

**Table 4:** Summary of linear regression analysis for six variables (child’s age at test, gender, *z*-scored SFOC, parental education level, parent’s use of spatial language, and free-play option) predicting Spontaneous Focus on Space.

Variable	*B*	SE *B*	*β*
Constant Age at test *Z*-scored SFOC Gender Parental education level Parent’s spatial language Free-play option Adjusted *R*^2^ *F*	−0.30 0.20 0.65 −0.11 −0.03 −4.59 0.17 0.72 13.70^**^	0.89 0.09 0.11 0.19 0.12 2.97 0.19	0.26^*^ 0.66^**^ −0.06 −0.03 −0.17 0.10	

*B, unstandardized regression coefficient; SE, standard error; *β*, standardized regression coefficient; ∆*R*^2^, difference in the proportion of variance explained*.

* *p < 0.05*,

** *p < 0.01*.

**Table 5:** Summary of linear regression analysis for six variables (child’s age at test, gender, *z*-scored SFOC, parental education level, parent’s use of spatial language, and free-play option) predicting Spontaneous Focus on Numerosity.

Variable	*B*	SE *B*	*β*
Constant Age at test *Z*-scored SFOC Gender Parental education level Parent’s spatial language Free-play option Adjusted *R*^2^ *F*	−0.38 0.05 0.81 −0.23 −0.02 −5.17 0.10 0.72 13.84^**^	0.94 0.10 0.12 0.20 0.13 3.15 0.21	0.07 0.78^**^ −0.13 −0.02 −0.18 0.06	

*B, unstandardized regression coefficient; SE, standard error; *β*, standardized regression coefficient; ∆*R*^2^, difference in the proportion of variance explained*.

** *p < 0.01*.

### Validity of Spontaneous Focus on Space as a Construct, and Its Relation to Spatial Math

#### Relation Between Spontaneous Focus on Space, Spontaneous Focus on Numerosity, and Proportional Reasoning Abilities

To evaluate the relation between child’s age, gender, parental education level, general memory performance (*z*-scored SFOC), tendencies to spontaneously focus on numerosity (*z*-scored SFON), tendencies to spontaneously focus on space (*z*-scored SFOS), and children’s performance on a proportional scaling math task, we conducted a regression with these six initial measures as independent variables and total error distance on the proportional scaling task as the dependent variable (the sum of the differences between the target location and the child’s denotation from all of the scaling trials). Together, this combined model significantly predicted children’s proportional scaling performance, *F*(6, 41) = 2.94, *p* = 0.02, adjusted *R*^2^ = 0.20. Within this combined model, only parental education level significantly predicted proportional scaling performance, *β*_parenteducationlevel_ = −0.28, SE = 4.58, *t*(41) = −2.10, *p* = 0.04 (Table 6). That is, the lower the education level of the parent, the larger the error distances produced by children on the scaling proportion task. To identify whether a child’s SFOS or SFON score was predictive of their subsequent performance on a proportional scaling math task, above and beyond general memory performance, age and parental characteristics, we conducted a selective step-wise regression model with total error distance on a proportional scaling task as the dependent variable (independent variables were entered in this order: child’s age at test as a continuous metric, gender, parental education level, *z*-scored SFON, *z*-scored SFOC, and *z*-scored SFOS). SFOS performance was the best predictor of proportional scaling performance, *F*(1, 46) = 11.27, *p* = 0.002, adjusted *R*^2^ = 0.18, ∆*R*^2^ = 0.20, *β*_SFOS_ = −0.44, SE = 3.45, *t*(46) = −3.36, *p* = 0.002. Additionally, parental education level significantly accounted for additional variance, *F*(1, 45) = 4.69, *p* = 0.04, adjusted *R*^2^ = 0.24, ∆*R*^2^ = 0.08, where *β*_SFOS_ = −0.39, SE = 3.38, *t*(45) = −3.06, *p* = 0.004, tolerance = 0.97, VIF = 1.03 and *β*_parentaleducationlevel_ = −0.28, SE = 4.43, *t*(45) = −2.16, *p* = 0.04, tolerance = 0.97, VIF = 1.03 (Table 3). In this step-wise regression model, child’s gender [*β*_gender_ = −0.06, *t*(45) = −0.43, *p* = 0.67, tolerance = 0.99, VIF = 1.01], age at test, [*β*_age_ = −0.18, *t*(45) = −1.19, *p* = 0.24, tolerance = 0.69, VIF = 1.43], general recall ability (SFOC) [*β*_SFOC_ = −0.06, *t*(45) = −0.27, *p* = 0.78, tolerance = 0.32, VIF = 3.16], and Spontaneous Focus on Numerosity (SFON) [*β*_SFON_ = −0.02, *t*(45) = −0.11, *p* = 0.92, tolerance = 0.40, VIF = 2.53] did not predict children’s proportional reasoning performance above and beyond their Spontaneous Focus on Space (SFOS) and Parental Education Level. Children who exhibited greater Spontaneous Focus on Space tendencies and who had more educated parents produced less errors on the proportional scaling task. However, because SFOS and SFON are highly correlated, we sought to do a more rigorous analysis identifying whether this finding could potentially be randomly driven by error, or the relatedness of SFOS and SFON, in the coding schemes. Children who catastrophically failed to Spontaneously Focus on Number (SFON of 0.25 or below) were removed. SFOS and SFON in this limited sample were still correlated (*r* = 0.64, *p* < 0.01, but less so than in the original sample *r* = 0.78, *p* < 0.01). To identify whether a child’s SFOS or SFON score was predictive of proportional scaling performance in this limited sample, independent variables were entered into a step-wise regression analyses in this order: child’s age at test as a continuous metric, gender, parental education level, *z*-scored SFON, *z*-scored SFOC, and *z*-scored SFOS. SFOS performance was the best predictor of proportional scaling performance [*F*(1, 37) = 12.41, *p* = 0.001, *β*_SFOS_ = −0.50, *t*(1, 37) = −3.5, *p* = 0.001]. However, unlike the previous model with the larger sample, no other variable predicted additional variability in proportional scaling performance. To further address which component of Spontaneous Focus on Space best predicted performance on the proportional scaling task, we conducted another analysis wherein Absolute SFOS (referring to the location placement of objects or stamps on certain parts of the scene or picture) and Relative SFOS (referring to the placement and orientation of objects or stamps to each other) were included as separate independent variables in this limited sample analyses. In this step-wise regression analyses, error distance on the proportional scaling task was included as the dependent variable, and independent variables were entered in this order: child’s age at test as a continuous value, gender, parental education level, *z*-scored SFON, *z*-scored SFOC, *z*-scored Absolute SFOS, *z*-scored Relative SFOS; Relative SFOS was the best predictor of proportional scaling performance [*F*(1, 37) = 10.88, *p* = 0.002, *β*_RelativeSFOS_ = −0.48, *t*(1, 37) = −3.3, *p* = 0.002] and no other factors significantly predicted performance. SFON did not predict proportional scaling performance, but SFOS did, specifically spontaneously focusing or relative space (the relations between entities), suggesting that successful completion of the proportional scaling task is more dependent on children’s spatial attention than numerical attention.

**Table 6:** Summary of linear step-wise regression analysis for six variables (child’s age at test, gender, parental education level, *z*-scored SFON, *z*-scored SFOC, and *z*-scored SFOS) predicting performance on the proportional scaling task.

Variable	*B*	SE *B*	*β*
**Model 1**			
Constant *Z*-scored SFOS Adjusted *R*^2^ *F*	49.21 −11.59 0.18 11.27^**^	3.23 3.45	−0.44^**^	
**Model 2**			
Constant *Z*-scored SFOS Parent education Level Age at test Child’s gender *Z*-scored SFON *Z*-scored SFOC ∆*R*^2^ *F*	92.06 −10.32 −9.59 −0.18 −0.06 −0.02 −0.06 0.08 4.69^*^	20.04 3.38 4.43	−0.39^**^ −0.28^*^	

*B, unstandardized regression coefficient; SE, standard error; *β*, standardized regression coefficient; ∆*R*^2^, difference in the proportion of variance explained*.

* *p < 0.05*,

** *p < 0.01*.

### Exploratory Analyses

#### Proportional Scaling Abilities Improve Throughout Early Development

**Figure 3:**
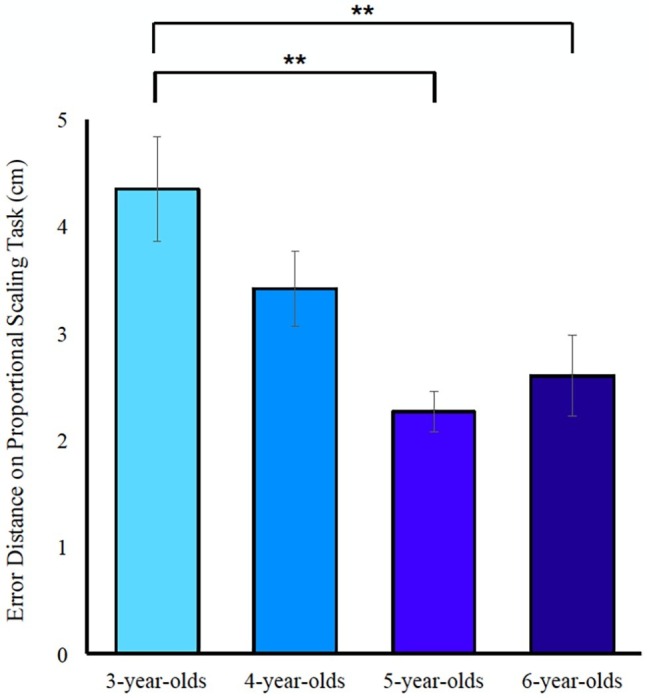
Paired-comparisons, Bonferroni adjusted for multiple comparisons, depict between-age group differences on children’s performance on the proportional scaling task, where errors produced decrease as age increases. Error bars reflect SEM. Asterisk symbols indicate significance at an alpha level *p* < 0.01.

The current design of this study allowed us to not only measure and validate Spontaneous Focus of Space alongside Spontaneous Focus on Numerosity from early in development, but to also compare proportional scaling abilities between 3-, 4-, 5- and 6-year-old children on a single, uniform task. To assess the developmental trajectory of proportional scaling abilities, we conducted a regression with absolute error distance (the sum of the distances from the correct answer for all scaling trials) on the proportional scaling task as the dependent variable, and age (as a continuous variable) as the independent variable. We found a significant improvement in scaling abilities with maturation, *F*(1, 53) = 10.29, *p* < 0.01, ∆*R*^2^ = 0.15. Within this model, child’s age at test was a significant predictor of proportional scaling performance in that older children made less errors on the proportional scaling task, *β*_age_ = −0.40, *t*(53) = −3.21, *p* < 0.01. Because we wanted to assess whether young children could engage in a proportional scaling task, we also conducted a one-way ANOVA with absolute error distance as the dependent factor and age at test as the independent factor so that we could look at parametric differences across age groups, *F*(3, 51) = 6.419, *p* < 0.01. *Post hoc* analyses with Bonferroni adjustments for multiple comparisons show a developmental trend in scaling improvement, where 5 and 6-year-olds significantly produce smaller errors than 3-year-olds [*M*_difference_ = 27.89, SEM = 8.36, *p* = 0.01, CI (4.95, 50.84) and *M*_difference_ = 33.22, SEM = 8.24, *p* < 0.01 CI (10.61, 55.83), respectively] and 3- and 4-year-olds do not significantly differ from each other nor do 5- and 6-year-olds (see Figure 3). These findings demonstrate that very young children can engage in and complete a proportional scaling task, and that performance on proportional scaling tasks parametrically increases with age. Because previous work has found stable individual differences in SFOS, we assessed the reliability of SFOS, SFON, and SFOC by first normalizing each measure (obtaining z-scores for each measure) and then calculating reliability statistics; Cronbach’s alpha for the three spontaneous focus measures was 0.94. Descriptive information about these three spontaneous focus measures (SFON, SFOC, and SFOS) and children’s proportional reasoning abilities is provided in Table 1 and the correlations between these variables in addition to parent’s use of spatial language are provided in Table 7.

**Table 7:** Correlation matrix (Pearson).

	SFON	SFOC	SFOS	Proportional Reasoning	Parental Spatial Language
SFON SFOC SFOS Proportional Reasoning Parental Spatial Language	—	0.88^**^ —	0.78^**^ 0.84^**^ —	−0.38^**^ −0.34^**^ −0.43^**^ —	−0.22 −0.08 −0.26 0.18 —

** *Correlation is significant at the 0.01 level (2-tailed)*.

#### Gender Differences in Free-Play Choice Activity

Further exploratory analyses show that there was a significant gender difference in the type of activity child–parent dyads chose to engage with during the free-play period of the study. Of the 36 children who responded to the free-play question, more boys and their parents (*M* = 0.63, SEM = 0.125) opted to engage in Lego^®^ building than did girls and their parents [*M* = 0.25, SEM = 0.10; *t*(34) = 2.38, *p* = 0.02]. Whether the child or the parent made the ultimate decision to play with the Lego © pieces or the crayons was not coded, but this gender difference speaks to an early emerging gendered bias in engaging in spatial tasks.

## Discussion

### Developmental Trajectory of Spontaneous Focus on Space

The purpose of this study was to develop a measure to assess young children’s attention to spatial relations (SFOS) alongside their attention to numerosity (SFON). Previous research has found that individual differences in the propensity to attend to numerosity are stable throughout early development, and increase with maturation (Hannula and Lehtinen, 2005). Although the current sample size is relatively small, we similarly find a developmental progression in SFON and SFOS abilities; older children demonstrate higher tendencies to focus on numerosity and on space than do younger children. These findings demonstrate that spatial awareness rapidly increases in the preschool years, alongside children’s spontaneous focus on numerosity.

Moreover, with the design of the current study, we had intended to separately investigate children’s general attention to, and memory for, non-quantity aspects of a scene (e.g., by measuring attention to color) in addition to their spontaneous focus on space and on numerosity. We found that children’s recall of color information significantly predicted SFON and SFOS performance. That is, a child’s attention to and recall of the color of entities in the scene predicted additional variance in their Spontaneous Focus on Numerosity and on Space tendencies above and beyond demographic factors like child’s gender, parental education level, and parent’s use of spatial language. Given the high correlation between our measures of SFOC, SFON, and SFOS, and the interplay between these measures when coding them, one possibility is that they are all the same underlying cognitive construct. However, we think they are psychologically separable for several reasons. First, if we restrict the dataset to include only those children who provided separable measures (e.g., placed only one target item, thus precluding the conflation of SFON and SFOS), the central findings hold. Second, the finding that SFOS, SFON, and SFOC differentially explain the variability in the proportional scaling tasks speaks against them measuring the same construct. Finally, the developmental trajectories for improvement in each of these measures was distinct, with SFOS performance improving significantly and steadily across 3–6 years of age, SFON improving from 5 to 6 years of age, and SFOC improving between 3 and 4 years of age.

Although we designed the measurement of SFOC to evaluate a child’s overall ability to attend to and recall non-magnitude information (e.g., color), our measurement is limited in that identifying and remembering the color of an object is intrinsically related to the spatial information and identity of that object. Since objects can be individuated both by their feature (what) and spatial (where) information (Kahneman et al., 1992), and spatiotemporal cues are known to be critical for how we perceive and remember objects in our environment (Flombaum and Scholl, 2006; Flombaum et al., 2009; Schurgin and Flombaum, 2017), it is possible that SFOC is not a pure measure of non-quantitative information. This likely is why studies of SFON in the past have sought to evaluate children’s general memory abilities using additional metrics like non-verbal IQ tasks or receptive vocabulary measures (Hannula and Lehtinen, 2005). In trying to produce a single task that captured children’s attention to multiple factors in a scene, we inadvertently overlooked how these factors must be intertwined to produce a percept of a set of individual objects. Future investigations on SFOS and SFON should aim to identify a measure that more rigorously evaluates a child’s general memory process on these tasks, that is unrelated to identifying the features of entities involved.

### Spontaneous Focus on Space and Proportional Scaling Abilities

Based on previous research which has shown the link between space and proportional reasoning (Jeong et al., 2007), we hypothesized that SFOS scores would predict proportional scale task error. When we controlled for age, gender, parental education level, SFON, and SFOC, SFOS significantly predicted performance on the proportional scaling task. Parent’s education level also accounted for additional variance in children’s’ scaling performance. Participants who exhibited more accuracy in their proportional scaling ability were better at recognizing and attending to the spatial characteristics of objects in a scene. Critically, SFON did not predict children’s performance on the proportional scaling task once general factors such as age and parent education were taken into account. We had theorized that although SFON is predictive of numeracy and arithmetic performance, it may not be central to reasoning about fractions and proportions, because young children find these concepts difficult unless they are presented in a highly spatialized and continuous manner. Indeed, the results from this study suggest that a measure of children’s spontaneous focus on space may be a better predictor of children’s ability to reason about proportions. Moreover, although spatial skills are highly related to mathematical skills, our results suggest that spatial and mathematical spontaneous foci are different mental constructs within the individual. As previous studies have shown, it is the individual variability in *spatial reasoning abilities* across various dimensions of spatial cognition that is predictive of performance in STEM-related disciplines like engineering (Hsi et al., 1997) and chemistry (see Wu and Shah, 2004 for a review). These findings support previous work investigating the relation between space and proportional reasoning (Jeong et al., 2007), and further suggest that although proportional reasoning is indeed mathematical, success on this computation may heavily rely on spatial cognition.

### Parental Factors and Spontaneous Focus on Space

We also hypothesized that SFOS scores would be positively related to parent spatial language. This was based on previous research which suggested that spatial language, even when task-irrelevant, promotes children’s success on spatial tasks (Pruden et al., 2011). In this study, we examined parent spatial language in order to examine the environment in which children are exposed to spatial thinking. However, when we controlled for age, general memory performance (SFOC), gender, parental education level, and the type of activity parent–child dyads chose to engage with before the experiment (a Lego© building task or coloring task), no relation between parent spatial language and SFOS score was found. Furthermore, the context in which children and parents engaged in spatial talk was unguided; parents were not prompted during the activity to specifically engage in spatial talk, but instead to simply engage in the activity with their child. Consequently, parents of older children who could independently complete the building task may have refrained from providing as much instruction, because the children did not need it. Moreover, because of the relatively small sample size in this study, future investigations with more children across each age group and with more parent-child dyad pairs within each age group may observe stronger predictive relations between parent spatial language use and child’s spontaneous focus on space, especially among younger children. Although we did not observe any predictive relation between parents’ use of spatial language on the building task that came before the Spontaneous Focus on Space tasks, in hindsight it would have been better to have this task come after all of the spontaneous imitation tasks and proportional reasoning tasks were completed to ensure that children could not have being primed by their parent before engaging in spatial and numerical tasks with the experimenter. Regardless, we did not find that children of parents who used a lot of spatial language fared better than those of parents who used a little spatial language, suggesting that SFOS performance was in fact measuring a child’s spontaneous focus on space irrespective of their prior interaction with their parent. Future studies may find stronger relations between SFOS and parental spatial language if the difficulty of the building task administered is normalized for child’s age at test (Wechsler, 1967).

Although we did not observe a relation between parental spatial language and children’s spontaneous focus on space, we did observe a relation between child’s gender and the activity parent–child dyads chose to engage in while the experimenter set up the study. That is, of the choice to build with Lego© pieces or to color with crayons, significantly more boy-parent dyads than girl-parent dyads chose Lego© building. Although allowing the parents and children to self-elect whether they wanted to engage in a Lego© building or coloring task could have offered some children more practice in thinking spatially before continuing to the spontaneous spatial and numerical tasks, we did not observe any predictive relation between which activity children engaged with during the free-play time and subsequent performance on SFOS and SFON. Further, after this free-play time, all children and their parents proceeded to complete a Lego© building task ensuring that all children were engaged in a spatial activity before the imitation trials began. We found no predictive relation between what children chose to play with and their subsequent SFOS performance, but, the current gendered findings mentioned previously are consistent with previous research which has found that gender differences in spatial skills emerge early in life (Levine et al., 1999; Pruden et al., 2011). More specifically, the current findings are consistent with previous literature that has investigated how sex typing activities purport spatial activities to be more suited for men over women, thus limiting the richness of women’s overall experience with spatial activities (Baenninger and Newcombe, 1989; Levine et al., 2012). And because experience with spatial activities like consistent puzzle play early in development is predictive of later spatial skills (i.e., spatial transformation; Levine et al., 2012), the current findings highlight the existence of a gendered difference in what boys and girls are encouraged to engage with even in informal learning situations like a children’s museum. This finding is especially interesting given the fact that it was *girls*, not boys, who had better scores overall for both spontaneous focus on numerosity and spontaneous focus on space.

Previous work investigating the development of spatial skills has found that maternal education level and the corresponding use of spatial language by highly educated mothers positively predicts children’s performance early spatial and mathematical tasks (i.e., performance on the spatial assembly 2-D and 3-D TOSA tasks in addition to the Number and Operations Subtest of the EMAS at age 3; Verdine et al., 2014a,b). Although our measure of parental-spatial language was limited given the independence afforded to older children during the Lego^®^ construction task, like previous studies (i.e., performance on the Wechsler Individual Achievement Test – Math Problem Solving subtest at age 4; Verdine et al., 2014a,b), we did observe a positive relation between parental education level and children’s mathematical performance. Moreover, in the current study, the dependent assessment of mathematical abilities was not focused on discrete problem-solving and instead assessed children’s proportional reasoning abilities (Möhring et al., 2015a,b), yet the positive relation between parental education level and mathematical performance was still observed. These findings reiterate the influence of parental education level on children’s early cognitive development (Hoff, 2003), but leave open which aspects of parental education level are the driving factors in preschoolers’ proportional reasoning abilities (i.e., perhaps it is due to the component of facilitating conversations that make use of spatial language; Hoff, 2003, 2006; Pruden et al., 2011; Verdine et al., 2014a,b).

The purpose of this study was to examine the developmental trajectory of children’s spontaneous focus on space, and its relation to SFON, and to proportional reasoning abilities. By looking at these relations, we gain insight into how children navigate their world through a detect, elect, connect framework, as shaped by a linguistics context and mathematical context (Perkins and Salomon, 2012). A strong measure of a child’s attention to spatial information is their propensity to *elect* to focus on spatial information in situations with other layers of engagement to draw their attention. They can then connect this spatial information to areas, such as formal mathematics, where it is ultimately useful. Therefore, considering the extent to which children actively elect to focus on spatial information in naturalistic and complex situations allows for the examination of a mechanism that could drive conceptual transfer. We observed a relation between spatial abilities (SFOS) and mathematical performance (here, proportional reasoning). Because of this observed relation early in childhood, it would be interesting to investigate whether SFOS may predict performance beyond mathematical reasoning, and whether it may serve as a predictor of future STEM performance like spatial orientation, spatial visualization, and/or mental rotation (Caldera et al., 1999; Wai et al., 2009; Levine et al., 2012; Mix and Cheng, 2012). Future research should explore the extent to which SFOS is predictive of performance in geometry or science. The current study speaks to the context in which children’s SFOS elicitation can be encouraged and facilitated. Informal environments such as museums are full of distraction and allow children to explore spatial concepts on their own, whereas children in school settings are guided to focus on the topic at hand. Yet, in both contexts, encouraging children to focus on the spatial relations between objects in the world around them may provide an earlier avenue through which spatial and mathematical abilities may be modifiable (Uttal et al., 2013). We believe the observed phenomena of SFOS, with its developmental progression and relation to mathematical performance, offers a tangible avenue for evaluating children’s spatial abilities. In summary, this study contributes a novel, nonverbal measure of spontaneous focus of space that does not conflate linguistic ability with spatial cognition, and that demonstrates continuous improvement throughout early development.

## Ethics Statement

This study was carried out in accordance with the recommendations of the Institutional Review Board, of Barnard College, Columbia University, with written informed consent from all subjects. All subjects gave written informed consent in accordance with the Declaration of Helsinki. The protocol was approved by the Institutional Review Board of Barnard College, Columbia University.

## Author Contributions

KM conceived of the presented idea. JP contributed to the design and implementation of the research. Both authors contributed to the analysis of the results and wrote the manuscript.

### Conflict of Interest

The authors declare that the research was conducted in the absence of any commercial or financial relationships that could be construed as a potential conflict of interest.
